# An Analytical Method for the Biomonitoring of Mercury in Bees and Beehive Products by Cold Vapor Atomic Fluorescence Spectrometry

**DOI:** 10.3390/molecules26164878

**Published:** 2021-08-12

**Authors:** Maria Luisa Astolfi, Marcelo Enrique Conti, Martina Ristorini, Maria Agostina Frezzini, Marco Papi, Lorenzo Massimi, Silvia Canepari

**Affiliations:** 1 Department of Chemistry, Sapienza University of Rome, Piazzale Aldo Moro 5, 00185 Rome, Italy; 2 Department of Management, Sapienza University of Rome, via del Castro Laurenziano 9, 00161 Rome, Italy; marcelo.conti@uniroma1.it; 3 Department of Bioscience and Territory, University of Molise, 86090 Pesche, Italy; m.ristorini@studenti.unimol.it; 4 Department of Environmental Biology, Sapienza University of Rome, Piazzale Aldo Moro 5, 00185 Rome, Italy; mariaagostina.frezzini@uniroma1.it (M.A.F.); l.massimi@uniroma1.it (L.M.); silvia.canepari@uniroma1.it (S.C.); 5 Association of Beekeepers of Rome and Province, via Albidona 20, 00118 Rome, Italy; buteo.betta@gmail.com

**Keywords:** bees, beehive products, biomonitoring, cold vapor atomic fluorescence spectrometry, sample preparation, toxic metal

## Abstract

Bees and their products are useful bioindicators of anthropogenic activities and could overcome the deficiencies of air quality networks. Among the environmental contaminants, mercury (Hg) is a toxic metal that can accumulate in living organisms. The first aim of this study was to develop a simple analytical method to determine Hg in small mass samples of bees and beehive products by cold vapor atomic fluorescence spectrometry. The proposed method was optimized for about 0.02 g bee, pollen, propolis, and royal jelly, 0.05 g beeswax and honey, or 0.1 g honeydew with 0.5 mL HCl, 0.2 mL HNO_3_, and 0.1 mL H_2_O_2_ in a water bath (95 °C, 30 min); samples were made up to a final volume of 5 mL deionized water. The method limits sample manipulation and the reagent mixture volume used. Detection limits were lower than 3 µg kg^−1^ for a sample mass of 0.02 g, and recoveries and precision were within 20% of the expected value and less than 10%, respectively, for many matrices. The second aim of the present study was to evaluate the proposed method’s performances on real samples collected in six areas of the Lazio region in Italy.

## 1. Introduction

Mercury (Hg) is an ubiquitous and toxic metal that continues to be a public health concern [1,2,3]. It is released into the environment from both natural and anthropogenic sources [4]. Mercury is present in the atmosphere as an elemental form (Hg^0^) and it is accumulated through the terrestrial and aquatic food webs as an organic form (methylmercury) [5,6]. Although Hg is associated with several adverse human health effects [7], it is still widely used in the chloralkali industry, for gold mining, and the production of dental amalgam, batteries, pesticides, fungicides, disinfectants, and antiseptics [8]. Because of the mentioned toxic properties, Hg monitoring in food and environmental samples is essential in order to perform reliable risk assessments and take appropriate actions to protect human health and the environment [9]. According to the current air quality directives in Europe, industrial activities must reduce Hg emissions by implementing control programs and integrated pollution prevention and, at the same time, by improving air quality assessment and monitoring programs [10,11,12,13]. Mercury in the atmosphere is mainly assessed by making punctual measurements with manual or automated air quality monitoring stations [14] and applying standardized methodologies, based on current legislation [10]. However, due to the high costs, the monitoring networks for Hg pollution assessment are still characterized by low temporal (generally on an annual basis) and spatial coverage [15]. For the above reasons, there is growing interest in alternative air monitoring techniques such as plant, insects, lichens, and mosses that can provide reliable time-integrated estimates of air pollution in a given area at low cost [16,17,18,19,20,21,22]. In particular, bees and their products such as honey, propolis, and pollen have been proposed as bioindicators of environmental Hg contamination [23,24,25]. The assessment of Hg levels in bee products is important not only for their use as possible bioindicators for environmental contamination purposes, but also for the potential human exposure due to their dietary, pharmaceutical, and cosmetic use [26,27,28,29,30].

Mercury has been studied in honey samples by several authors [24,25,31,32,33,34,35,36,37,38,39,40,41,42], whereas limited literature data are available regarding the Hg determination in beeswax [42], pollen [24,25,39,41,43,44], propolis [24,45,46,47], and bees [24,25,31,39,47,48,49]. Microwave-assisted digestion is the most commonly used technique for preparing bee samples and hive products [32,35,42,43,44]. However, the microwave-assisted digestion method requires certain sample masses and reagent volumes, often leading to high final dilution factors and a consequent increase in the method detection limits [25,50]. In contrast, some authors have miniaturized digestion of honey, pollen, and/or bees by heating them in a heat block (80–100 °C) and using very small reagent volumes [25,33]. Throughout the literature, many studies have quantified Hg concentrations in bees and beehive product matrices with atomic absorption spectroscopy [38] and inductively coupled plasma–mass or optical emission spectrometry (ICP-MS or ICP-OES, respectively) [25,32,40,41,42,43,49], often coupled to cold vapor generation (CV) for matrix separation [24,33,34,35,37,44,45], electrothermal atomic absorption spectrophotometry [47], and direct Hg analysis using automated commercial instruments such as the advanced mercury analyzer (AMA) [36,39,46] or direct mercury analyzer (DMA) [37,48]. CV atomic fluorescence spectrometry (AFS) is a good alternative for total Hg determination, and has been commonly employed for the analysis of several biological and environmental matrices [51], food [52], and human bodily fluids and tissues [53,54,55]. Despite this, CV-AFS has rarely been applied for the determination of Hg in honey [35], and, to the best of our knowledge, this technique has not been applied in bees and other beehive products.

This study aimed to miniaturize the sample digestion of bees and beehive products to achieve accurate and reproducible results with low detection limits for Hg determination by CV-AFS. The proposed analytical method was applied to commercial honeydew and royal jelly samples and bees, honey, beeswax, pollen, and propolis samples collected from six central Italy areas were characterized by different exposure to environmental pollution.

## 2. Results and Discussion

### 2.1. Comparison with Previous Methods

The analytical characteristics comparison of the method proposed in the present study with others already developed for Hg determination in bee and beehive product samples is shown in Table S1. In this study, the sample digestion was miniaturized by reducing all volumes and masses to allow sample preparation in one disposable test tube. This prevented sample loss due to the transfer in different tubes and minimized possible contamination. In addition, the use of smaller volumes of reagents allows for a lower final dilution factor (5×), and lower method detection limit (DL) and decreases the consumable and chemical waste generated, meeting the ever-increasing demand to comply with green chemistry requirements. The dilution factor, together with the sample mass, the reagent purity, and the chosen instrument, can affect the Hg DL in bees and beehive products, where this metal is generally present in low concentrations. To decrease the method DL, the sample mass can be increased, but sometimes this is not possible (such as for bees or specific pollen). Furthermore, if the analytical method requires sample digestion, the increase in sample mass must necessarily be accompanied by an appropriate volume of reagents to ensure complete sample digestion. Even for methods that do not require sample pre-treatment such as AMA or DMA (Table S1), the sample mass cannot be randomly increased to ensure complete drying, ashing, and atomization of the sample. In addition, amounts larger than 100 mg of sample can produce a build-up of combustion gases, resulting in a rapid increase of pressure in the furnace [37]. In the literature (see Table S1), some studies have used a large final dilution of bees and beehive product samples (25–50×) [24,33,35,36,40,41,44,47,49] to reduce the acidity of the final digest, sometimes compromising the Hg determination. For this purpose, in this study, various sample aliquots (0.05–1 g for honey and honeydew, 0.02–0.2 g for bees, beeswax, pollen, propolis, royal jelly) were digested at the maximum temperature of 95 °C, considering two different times (30 or 60 min) and using the smallest amount of reagent mixture (0.5 mL HCl, 0.2 mL HNO_3_, and 0.1 mL H_2_O_2_) to employ the smallest dilution factor final (5×). The choice of acid and oxidizing agent (HNO_3_ and H_2_O_2_, respectively) is widely agreed by most of the literature for the selected matrices [24,25,33,35,36,38,41,42,44,47,48,49], while HCl was selected according to the manufacturer’s recommendations.

The proposed sample preparation also appears to be the fastest procedure (digestion time, 30 min for 120 samples or more) compared to the other sample treatments reported in the literature [25,33,40,41,42,44,47] (Table S1), resulting in being suitable for routine analysis with high sample throughput and biomonitoring. However, it should be noted that possible volatile Hg species such as organometallic compounds or metal nanoparticles could be lost during digestion due to their volatilization.

The analytical characteristics of the proposed method are detailed in the following sections.

### 2.2. Linearity and Selectivity

The linearity and selectivity of the proposed method were evaluated by preparing calibration curves in aqueous [3% (*v*/*v*) HCl and HNO_3_] standards and using the standard addition method at Hg concentrations of 0.00, 0.02, 0.04, 0.1, 0.2, 0.4, 0.8, 1.0, and 1.5 µg L^−1^. Digested samples of each matrix (20 mg) were diluted to reach the same acid ratio as the aqueous standard solutions and used to create calibration curves with the standard addition method. The linearity ranges from 0.02 to 1.5 µg L^−1^ were checked through the linear regression coefficient (R^2^) and verified by the Mandel fitting test. Calibration curve points with percent relative deviation ≥10% from calculated concentrations were tested and removed using the instrument software. The parameters of the calibration curves after the outliers’ removal are presented in Table 1. Data of the calibration curves using aqueous standards were obtained by nine independent replicates. The dynamic range was compared with that of other previously published methods (Table S1). In particular, CV-AFS allows for the determination of Hg in a wide range of concentrations, showing a dynamic range greater than that possible with other techniques such as CV-AAS, ICP-OES, and DMA. The matrix effect was evaluated by comparing the slopes of the calibration curves obtained from aqueous standards and the standard addition method (Table 1). Most of the results showed good data dispersion; however, some standard deviation values were of the same order of magnitude as the intercept data, generating a large statistical uncertainty on these data. The *t*-test at a 95% confidence level was used to evaluate possible significant differences between the angular coefficients of the calibration curves, in accordance with previous studies [25,37,56]. There were no apparent matrix effects between the aqueous and standard addition calibration curves. Thus, these results agree with those obtained for bees, honey, and pollen by other authors [25,37].

### 2.3. Detection and Quantification Limits

The DL was calculated based on the calibration curve using software prepared by the Regional Agency for Environmental Protection [57]. Therefore, the DL can be expressed as DL = 3.3 σ/b; where the coefficient 3.3 is called the expansion factor and is obtained assuming a 95% confidence level; σ is the standard deviation of the response of the curve; and b is the calibration curve slope. The reached DL of 0.01 µg L^−^^1^ for aqueous calibration confirmed the excellent sensitivity of the proposed method. The QL was set at the lowest standard curve points of calibration, which was 0.02 µg L^−^^1^. The DL and QL varied depending on the mass of the analyzed matrix and dilution required before analysis (in this study, 5×). In particular, for a mass of 0.02, 0.05, 0.1, 0.2, and 1 g, the DL was 3, 1, 0.5, 0.3, and 0.05 μg kg^−^^1^, and the QL was 5, 2, 1, 0.5, 0.1 μg kg^−^^1^, respectively. As shown in Table S1, the obtained DLs are comparable to previously reported AMA or DMA analysis [36,37,39] and ICP-MS analysis [25] and lower than CV-AAS or CV-ICP-OES analysis [24,33].

### 2.4. Accuracy and Precision

Due to the lack of certified reference material of bees and beehive products, the accuracy and precision (as repeatability and intermediate precision) of the proposed method were evaluated by recovery tests in agreement with other authors [25,36,37] and as indicated by Commission Decision no. 657/2002 [58]. Samples of each matrix (0.05–1 g for honey and honeydew, 0.02–0.2 g for bees, beeswax, pollen, propolis, royal jelly) were spiked with Hg at low (0.02 µg L^−1^), intermediate (0.2 µg L^−1^), and high (1 µg L^−1^) concentration and then digested. The method performance at levels near the QL was evaluated considering the smallest mass of each matrix and the shortest digestion time (30 min). The same solutions were again analyzed on two separate days to assess intermediate precision. The recovery and precision (such as repeatability) data are shown in Table 2 and Tables S1 and S2. Intermediate precision data (not shown) were very similar to the repeatability values.

In summary, the digestion time and mass for each matrix suitable for obtaining recoveries and precision within 20% of the expected value and less than 10%, respectively, were tabulated (Table 3). In this study, the major sources of uncertainty were the recovery of the procedure, instrumental calibration, and repeatability of the measurements. In contrast, the samples’ weights were the lowest contribution to the Hg uncertainty, in agreement with a previous study [59].

### 2.5. Hg Concentrations in Real Samples

**Figure 1:**
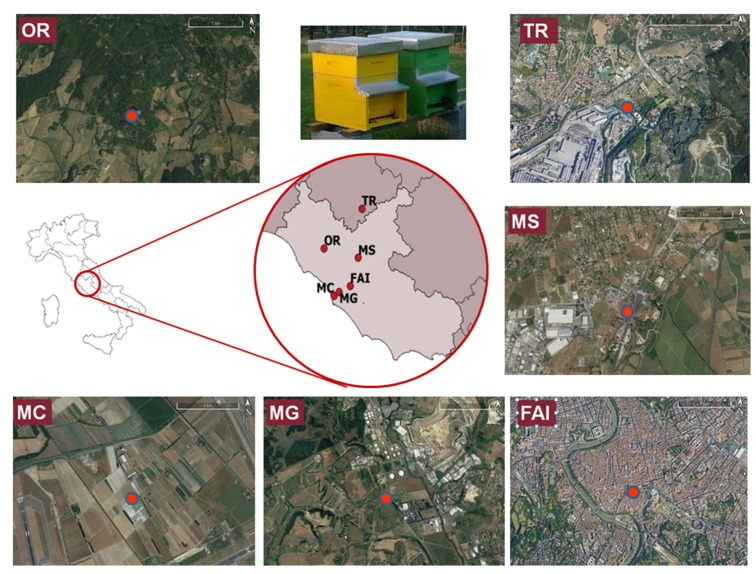
Geographic location of sampled apiaries (central Italy).

Bees and beehive products (honey, beeswax, pollen, and propolis) from various geographical areas in central Italy (Figure 1) and commercial samples of both honeydew and royal jelly were analyzed to demonstrate the applicability of the proposed method for routine analysis and biomonitoring.

Mercury pollution is an important environmental and public health issue. Elemental Hg can be emitted into the atmosphere by both anthropogenic (mainly artisanal gold mining, fossil fuel combustion, and cement production) and natural (such as a geothermal activity) sources [39]. Subsequently, Hg is transported to land and surface waters through wet and dry deposition, where it can undergo a bioconversion into more volatile or soluble forms such as methylmercury and return into the atmosphere or bioaccumulate in food chains [39]. Additionally, bees are continuously exposed to contaminants including Hg. Every day during foraging activities, bees gather nectar, plant resins, and water in the border of 7 km^2^ around their beehive and may come into contact with chemicals [47,49]. Therefore, the bee was proposed as a multi-sample contaminant collector because of its high mobility, contact with possible chemicals through inhalation, digestion, and hairs covering its body [40,47,49]. Contaminants adhered to the hairs such as particles of soil and dust can be carried into the beehive, thus affecting the composition of the beehive products [42,49]. In addition, Hg captured by the leaves of plants or absorbed from the soil through the plant root system can influence the nectar and pollen composition, which are brought back into the beehive [42,44]. Furthermore, propolis, produced with plant resin and mixed with salivary secretions and wax, due to the sticky nature of gum, might be used as a bioindicator of atmospheric pollution [46,47].

These considerations form the basis with which bees and their products have been proposed as reliable bioindicators of the environment including the atmosphere and pollution [40,42,43,46,47,48,49,60].

The Hg levels in all royal jelly samples were lower than DL, while in honeydew samples, they were 0.83 ± 0.34 µg kg^−1^. As shown in Table 4, the Hg concentrations were above the DL for many matrices, showing that the proposed method can be used to determine the Hg level in bees and beehive products. Although variation in Hg level across different areas for each matrix indicates the possibility of using the proposed method for biomonitoring, alternative and parallel measurements of the contamination of the environmental compartment of interest are necessary.

For honey, the mean Hg concentrations in this study (0.91–3.37 µg kg^−1^) were in agreement with the mean contents found in Croatia (0.47–0.52 µg kg^−1^) by Bilandžić et al. [36] and China (0.34–4.00 µg kg^−1^) by Ru et al. [35]. In another Italian study, Hg levels were lower than the quantification limit of 2 µg kg^−1^ [32]. The mean Hg levels in pollen (3.2–12.8 µg kg^−1^) were similar to the concentrations reported in Poland (3.6–6.6 µg kg^−1^) by Roman [43] and in Brazil (0.4–6.8 µg kg^−1^) by Morgano et al. [44]. According to our mean data in propolis (4.6–14.8 µg kg^−1^), studies from Croatia by Cvek et al. [45] and Spain by Bonvehí and Bermejo [46] reported Hg concentrations as a median of 12 µg kg^−1^ and mean of 8.0 ± 2.5 µg kg^−1^, respectively. For bees, there are few available data in the literature because of the limited amount of this matrix and consequently high DL values [24,25,40,47,48]. A study by Toth et al. [39] reported Hg concentrations of 39.892 ± 0.035 µg kg^−1^ and 8.224 ± 0.028 µg kg^−1^ in bees from two locations in eastern Slovakia. These results are in accordance with our data ranging from 0.53 to 31 µg kg^−1^. For beeswax, only one study by Bommuraj et al. [42] reports a concentration value of Hg equal to 62 µg kg^−1^, while our data fell in the range of <1–12.7 µg kg^−1^. Unfortunately, it was not possible to make a comparison with the literature for honeydew and royal jelly.

In this study, the Hg levels showed a typical distribution related to anthropogenic development of the areas. Furthermore, in agreement with the observations of other authors on the biological barrier capacity of bees for the contamination of honey by Cd and Tl [40], bees also seem to work as biofilters for Hg. In fact, the Hg levels were generally lower in the rural site (OR) and honey samples and higher in the sites with greater anthropogenic impact and bee samples. In particular, bees showed approximately ten times higher mean concentrations in the FAI (16.2 ± 2.7 µg kg^−1^), MG (16.2 ± 5.6 µg kg^−1^), and MS (17.8 ± 8.5 µg kg^−1^) areas than in the OR site (1.76 ± 0.85 µg kg^−1^). The lowest Hg level was detected in honey samples from OR (0.91 ± 0.23 µg kg^−1^). The principal anthropogenic sources of Hg pollution are industrial and urban discharge and combustion [35,38].

Our results agree with numerous other studies [60,61]. In Toth et al. [39], a statistically significant relationship was described between the locality and Hg content in bees and bee pollen. Moreover, in the study by Dżugan et al. [40], the sampling area and its related emission sources influenced toxic metal concentration in both bee bodies and honey. However, the Hg content in bees may also depend on other factors such as method of rearing bee colonies (including supplemental feeding), age of worker bees, and physiological and health status of bee specimens and bee colonies [62]. Due to its physical feature (sticky) and its chemical composition (mainly polyphenols, amino acids, terpenes, and steroids), propolis can absorb Hg and other metals [47,63], thus it can also be used as a bioindicator of air pollution [63,64].

Especially for honey, the assessment of Hg levels is important not only for environmental protection but also for food quality and consumer health [38]. Currently, the Hg presence in honey must not follow specific regulations. However, the Codex Alimentarius states that honey shall be free from metals in amounts that may result in a hazard to human health [65]. A provisional tolerable weekly intake (PTWI) of 0.3 mg (0.042 mg/day) for a 70-kg person (0.004 mg/kg body weight/week) was designed for Hg [66]. Considering the highest Hg concentration of the whole campaign (3.80 µg kg^−1^), a 20-g daily honey consumption represents a weekly intake of circa 0.2% of the PTWI for Hg. This Hg intake is well below the recommended dose, and the consumption of honey is not considered dangerous for human health.

## 3. Materials and Methods

### 3.1. Study Areas and Sample Collection

Samples of royal jelly (*n* = 2) and honeydew (*n* = 2) of different brands were purchased in duplicate from the Italian market, while samples of bees, beeswax, honey, pollen, and propolis were collected from six different apiaries across central Italy from April 2018 to June 2019 (Figure 1). Two beehives were selected at each apiary, and the beekeepers sampled their bee colonies and beehive products into polyethylene screw-cap containers once every two months in the late morning. The six study locations were chosen to represent sites with different human activities and environmental impacts. Terni (TR) was selected as an industrial area affected by the steel mill industry. Rome [city center on the roof of the Apicultural Italian Federation (FAI), Anagnina (MS), Malagrotta (MG), and Maccarese (MC)] was chosen as an urban area influenced by different emission sources such as traffic pollution in FAI and MS; biomass burning in FAI; various industrial plants such as refinery, gasifier, hospital waste incinerator, landfill of municipal waste, and quarries for the extraction of building materials in MG; and intense air and ship traffic in MC (located next to Fiumicino airport). Finally, Oriolo Romano (OR) in Viterbo province was selected as a rural area.

After sampling, the samples were transported to the laboratory. For each beehive, bees (*n* = 20) were dried in a freeze drier (at least 48 h for constant weight) and then were ground in a ceramic mortar coated with parafilm. Beeswax samples were separated from honey, washed in deionized water until all of the residual honey was removed, and dried using a freeze dryer (at least 24 h for constant weight). All of the obtained samples were thoroughly mixed to have a homogeneous sample and were stored at −18 °C until analysis.

### 3.2. Materials and Reagents

Certified Hg standard solution of 1002 ± 7 mg L^−1^ in 10% HNO_3_ was obtained from SCP Science (Baie D’Urfé, Quebec, Canada) and was used for further dilutions in order to prepare eight calibration standard solutions in the range from 0.02 to 1.5 µg L^−1^. HNO_3_ (67%, suprapure), HCl (30%, suprapure), NaOH (98%, anhydrous pellets, and RPE for analysis, ACS–ISO) were purchased from Carlo Erba Reagents (Milan, Italy) and H_2_O_2_ (30%, suprapure) and NaBH_4_ were obtained from Merck KgaA (Darmstadt, Germany). Deionized H_2_O (resistivity, ≤18.2 MΩ cm) from an Arioso Power I RO-UP Scholar UV system (Human Corporation, Songpa-Ku, Seoul, Korea) was used throughout the study.

Graduated tubes (2.5, 5, and 10 mL in polypropylene) were purchased from Artiglass S.R.L. (Due Carrare, PD, Italy), and syringe filters (0.45-μm pore size and cellulose nitrate membrane) were obtained from GVS Filter Technology (Indianapolis, IN, USA).

### 3.3. Sample Preparation and Analysis

Preliminary experiments were conducted to optimize the sample digestion using a water bath (WB12, Argo Lab, Modena, Italy) at 95 °C and ~1 bar. A freeze dryer (Heto Power Dry LL1500, Thermo Electron Corporation, Waltham, USA) was employed with a vacuum of 10^−3^ mbar and a condensing plate temperature of −40 °C to dry the beeswax and bee samples. An aliquot of the samples (0.05–1 g for honey and honeydew, 0.02–0.2 g for bees, beeswax, pollen, propolis, royal jelly) was treated with 0.5 mL HCl, 0.2 mL HNO_3_, and 0.1 mL H_2_O_2_ into open graduated tubes for 30 or 60 min under a fume hood. Digestion blanks (*n* = 10) were carried out in the same way. All solutions of the digested samples were colorless and without suspended solid particles except for the honey, honeydew, and propolis solutions obtained from digestion of the largest mass. Thus, digested samples were diluted to a final volume of 5 mL with deionized water, filtered, and then analyzed with an AFS 8220 (Beijing Titan Instrumental Co. Ltd., Beijing, China) with Ar (99.999% purity, SOL Spa, Monza, Italy) as a carrier gas. HCl (5%, *v*/*v*) was used as a carrier liquid, and 2% (*w*/*v*) NaBH_4_ in 0.5% (*w*/*v*) NaOH was used as a reducing agent. The instrumental optimized parameters were previously described [59]. Duplicate analyses were performed for each sample. Blanks and control standards (at 0.4 µg L^−1^) were run every 20 determinations to evaluate instrument drift.

### 3.4. Quality Assurance

The analytical performance parameters of selectivity, linearity, detection and quantification limit (DL and QL, respectively), precision, and accuracy were evaluated. The validation process was performed using spiked real sample assays. Method blanks, in-house quality control samples, and spiked and non-spiked real samples (three replicates each) were prepared along with every digested sample batch. Hg standard solution at 2, 20, or 100 μg L^−1^ was made for spikes; 0.05 mL of the spike solution was added to appropriate tubes 30 min before reagents and digestion. At the instrument, the concentration was 0.02, 0.2, or 1 μg L^−1^. For the recovery determination, the non-spiked real sample concentration was subtracted from that measured in the spiked real sample.

An eight-point calibration curve consisting of Hg concentrations between 0.02 and 1.5 μg L^−1^ was prepared using aqueous standards and the standard addition method for each matrix. The DL was defined as the Hg concentration corresponding to three times the standard deviation of the digestion blanks (*n* = 10).

### 3.5. Statistical Analysis

Statistical analysis was performed using the SPSS 25.0 program (IBM Corp., Armonk, NY, USA). All data were normally distributed as confirmed by the Kolmogorov–Smirnov test. One-way ANOVA, followed by Bonferroni post-hoc test, was used to determine the significant differences among the Hg concentrations for each matrix in different geographical areas. The probability level of *p* < 0.05 was considered statistically significant. For statistical analysis, in samples where the Hg concentration was below DL, the used values were one half of DL.

## 4. Conclusions

Coupling water bath digestion with CV-AFS analysis proved to be a good analytical tool for evaluating Hg contamination in bees and beehive products (beeswax, honey, honeydew, pollen, propolis, and royal jelly). Due to the possibility of preparing the sample using same single autosampler tubes, the optimized digestion procedure allows for the prevention of sample loss, minimize manipulation, and reduce both the reagent volumes and final dilution. The proposed method is suitable for small masses (down to 0.02 g) of all selected matrices and can be used for biomonitoring and food quality control. In particular, the results from the application in the field of the proposed method showed a higher Hg concentration in bees than the other matrices considered and in areas with a higher anthropogenic impact than the background site. In the future, considering alternative and parallel measurements of the contamination of the environmental compartment of interest, it will be interesting to evaluate whether bees and hive products can indeed be used to assess environmental spatial changes in Hg levels. However, the determination of Hg concentrations in beehive products is also important for potential human dietary exposure. The Hg concentrations in the analyzed samples of honey, honeydew, and royal jelly are not a cause for concern for consumer health effects. Furthermore, the data in this study can be used as a reference for comparing Hg concentrations to other countries in the world.

## Figures and Tables

**Table 1:** Comparison of calibration curve parameters for Hg determination by cold vapor atomic fluorescence spectrometry (CV-AFS).

Calibration Standards	Parameter ^a^				
	a	s(a)	b	s(b)	R^2^
Aqueous standards	9.68 × 10	9.68 × 10	1.86 × 10^4^	1.16 × 10^3^	0.999
Bee-addition standards	1.32 × 10^2^	3.14 × 10	1.64 × 10^4^	2.30 × 10^3^	0.999
Beeswax-addition standards	7.65 × 10	1.05 × 10	1.71 × 10^4^	2.22 × 10^3^	0.999
Honey-addition standards	1.07 × 10^2^	1.34 × 10^2^	1.61 × 10^4^	2.05 × 10^3^	0.999
Honeydew-addition standards	1.38 × 10^2^	8.58 ×10	1.65 × 10^4^	1.78 × 10^3^	0.999
Pollen-addition standards	1.30 × 10^2^	3.73 × 10	1.62 × 10^4^	2.07 × 10^3^	0.998
Propolis-addition standards	3.85 × 10^2^	2.50 × 10^2^	1.76 × 10^4^	1.40 × 10^3^	0.999
Royal jelly-addition standards	7.65 × 10	1.05 × 10	1.80 × 10^4^	9.19 × 10^2^	0.998

^a^ a, intercept; s(a), standard deviation of intercept; b, slope; s(b), standard deviation of slope; R^2^, correlation coefficient.

**Table 2:** Recovery and precision data for Hg in bees and beehive products (*n* = 3) by water bath digestion (95 °C, 30 min).

		Low Level Spike (0.02 µg L^−1^)		Intermediate Level Spike (0.2 µg L^−1^)		High Level Spike (1 µg L^−1^)	
Matrix	Mass (g)	R%	CV%	R%	CV%	R%	CV%
Honey	0.05	86	5.4	116	9.3	96	7.8
Honeydew	0.1	89	0.4	113	10	91	8.4
Pollen	0.02	92	9.6	90	3.7	95	3.6
Propolis	0.02	104	9.8	98	8.6	91	2.5
Beeswax	0.05	92	10	111	8.5	99	2.0
Royal Jelly	0.02	117	9.3	108	4.4	110	0.9
Bees	0.02	95	8.9	97	4.5	91	10

**Table 3:** Summary of mass and digestion time that can be used in bees and beehive products.

Matrix	Mass ^a^ (g)	Digestion Time ^a^ (min)
Bees	0.02–0.2	30 or 60
Beeswax	0.02	60
	0.05–0.1	30 or 60
	0.2	60
Honey	0.05	30 or 60
	0.1	60
Honeydew	0.05	60
	0.1	30 or 60
	0.2	60
Pollen	0.02–0.2	30 or 60
Propolis	0.02	30 or 60
Royal Jelly	0.02–0.2	30 or 60

^a^ The method performance at levels near the QL (0.02 µg L^−1^) was evaluated considering the smallest mass of each matrix and the shortest digestion time (30 min).

**Table 4:** Comparison of mercury occurrence (µg kg^−1^) in six sampled apiaries (central Italy).

Matrix	Statistics	OR	FAI	MC	MG	MS	TR
Honey	N	14	6	10	10	10	4
	Mean	**0.91 ^a,b,c,d,e^**	**2.26 ^a^**	**2.68 ^b^**	**2.66 ^c^**	**2.43 ^d^**	**3.37 ^e^**
	SD	0.23	0.69	0.75	0.36	0.60	0.60
	Median	0.78	1.92	2.97	2.53	2.07	3.37
	Minimum	0.66	1.80	1.35	2.30	1.95	2.95
	Maximum	1.25	3.06	3.17	3.23	3.20	3.80
Pollen	N	14	NS	12	10	4	4
	Mean	**3.2 ^a,b^**	-	7.6	7.5	**12.8 ^a^**	**10.4 ^b^**
	SD	1.4	-	2.0	1.9	8.0	2.5
	Median	3.0	-	7.2	7.0	12.8	10.4
	Minimum	<3	-	5.1	5.9	7.2	8.7
	Maximum	5.6	-	10.2	10.6	18.5	12.2
Propolis	N	10	NS	NS	4	6	NS
	Mean	**4.6 ^a^**	-	-	**7.54 ^b^**	**14.8 ^a,b^**	-
	SD	1.2	-	-	0.65	2.1	-
	Median	4.8	-	-	7.54	15.7	-
	Minimum	<3	-	-	7.08	12.4	-
	Maximum	5.7	-	-	8.00	16.4	-
Beeswax	N	14	8	14	12	12	4
	Mean	**2.8 ^a^**	5.9	6.4	4.8	4.9	**11.5 ^a^**
	SD	1.6	2.8	3.0	1.7	3.1	1.8
	Median	2.8	5.2	4.5	4.2	3.9	11.5
	Minimum	<1	3.5	3.3	3.5	3.0	10.2
	Maximum	5.7	9.5	10.6	8.1	11.2	12.7
Bees	N	14	6	14	14	10	4
	Mean	**1.76 ^a,b,c^**	**16.2 ^a^**	11.0	**16.2 ^b^**	**17.1 ^c^**	14.5
	SD	0.85	2.7	5.8	5.6	8.5	5.2
	Median	2.09	15.1	10.4	14.5	17.0	14.5
	Minimum	0.53	14.4	1.6	8.3	9.1	10.9
	Maximum	2.65	19.3	20.3	25.3	31.0	18.2

N, samples number; SD, standard deviation; NS, not sampled. ^a,b,c,d,e^. The data in bold with the same superscript letters within rows were significantly different (*p* < 0.01; ANOVA test).

## Data Availability

The data presented in this study are available on request from the corresponding author.

## Supplementary Materials

The following are available online. Table S1: Summary of the analytical characteristics of the proposed method and comparison with some of the previous methods published during the last decade (2010–2020), Table S2: Recovery and precision data for Hg in bees and beehive products by water bath digestion (95 °C, 30 min or 60 min).

## Sample Availability

Some samples used in this study are available from the authors.
