# Supplementary material for: An Analytical Method for the Biomonitoring of Mercury in Bees and Beehive Products by Cold Vapor Atomic Fluorescence Spectrometry
Source: Molecules. 2021 Aug 12;26(16):4878. doi: 10.3390/molecules26164878 (PMC8399589; doi:10.3390/molecules26164878)
Supplement: Supplementary file 1 [file molecules-26-04878-s001.zip › molecules-1233766-supplementary.pdf]

Supplementary Material

An Analytical Method for the Biomonitoring of Mercury in Bees and Beehive Products by Cold Vapor Atomic Fluorescence Spectrometry

Maria Luisa Astolfi[1,*], Marcelo Enrique Conti[2], Martina Ristorini[3], Maria Agostina Frezzini[4], Marco Papi[5], Lorenzo Massimi[4], Silvia Canepari[4]

[1] Department of Chemistry, Sapienza University of Rome, Piazzale Aldo Moro 5, 00185 Rome, Italy;
[2] Department of Management, Sapienza University of Rome, Via del Castro Laurenziano 9, 00161 Rome, Italy; marcelo.conti@uniroma1.it (M.E.C.)
[3] Department of Bioscience and Territory, University of Molise, 86090 Pesche, Italy; m.ristorini@studenti.unimol.it (M.R.)
[4] Department of Environmental Biology, Sapienza University of Rome, Piazzale Aldo Moro 5, 00185 Rome, Italy; mariaagostina.frezzini@uniroma1.it (M.A.F.); l.massimi@uniroma1.it (L.M.); silvia.canepari@uniroma1.it (S.C.)
[5] Association of Beekeepers of Rome and Province, Via Albidona 20, 00118 Rome, Italy; buteo.betta@gmail.com (M.P.)
* Correspondence: marialuisa.astolfi@uniroma1.it; Tel./Fax: +39-06-4991-3384

Table S1 - Summary of the analytical characteristics of the proposed method and comparison with some previous methods published during the last decade (2010-2020).

Reference	Sample matrix	Sample preparation	Total sample digestion time	Technique	LOD	LOQ	Accuracy (R%)	Precision (CV%)	Dynamic range (Log)
This study	Bees, honey, beeswax, honeydew, pollen, propolis, and royal jelly	0.02 g bee, pollen, propolis and royal jelly, 0.05 g beeswax and honey, or 0.1 g honeydew + 0.5 mL HCl, 0.2 mL HNO_3 and 0.1 mL H_2O_2 were digested in a water bath (95 °C, 30 min) → volume completed to 5 mL deionized water.	About 30 min for 120 samples or more.	CV-AFS	0.5 µg kg^{-1} (for a sample mass of 0.1 g)–3 µg kg^{-1} (for a sample mass of 0.02 g)	1 µg kg^{-1} (for a sample mass of 0.1 g)–5 µg kg^{-1} (for a sample mass of 0.02 g)	86 (honey, 0.05 g)–117% (royal jelly, 0.02 g)	<10%	1.9
[1]	Bees, honey, and pollen	20–200 mg bee, 20–100 mg pollen or 50–200 mg honey + 0.2 mL HNO_3 followed by 0.1 mL H_2O_2 in a digestion block (80 °C, 2 h total); samples were made up to a final volume of 6 mL.	2h	ICP-MS	14, and 4 µg kg^{-1} for a honey sample mass of 0.05 and 0.2 g, respectively	-	96 (B)–129% (As) for trace elements and 91 (Pr)–112% (La) for rare earth elements	<15%	-
[2]	Bees, honey, pollen, and propolis	For the bee samples drying at 100 °C for 48 h preceded microwave digestion. 1g honey, and pollen, 0.25 g propolis, or 0.1 g bees dried weight (at 100 °C for 48 h) + 5 mL HNO_3 and 2.5 mL H_2O_2 were digested in the microwave oven (30 min) → volume completed to 25 mL	About 30 min	CV-AAS	50 µg kg^{-1} for honey, 60 µg kg^{-1} for pollen, 300 µg kg^{-1} for propolis and 800 µg kg^{-1} dw for bees	-	64 (propolis)–129% (honey)	<13%	0.6
[3]	Honey	0.1–2 g + 0.5 mL HNO_3 and 0.5 mL H_2O_2 → the mixture allowed to stand for 12 h → heated to 100 °C in a metal block for 3 h → volume brought to 25 mL by adding 0.5 mol L^{-1} HCl.	15 h	Cloud point extraction (CPE), CV-ICP-OES or ICP-OES	2 µg kg^{-1} (CPE CV-ICP OES)–300 µg kg^{-1} (ICP-OES)	-	R% close to 100% when up to 2.0 g of honey were subjected to sonication or up to 1.0 g of honey was decomposed with acid.	-	0.5 (ICP-OES)-1.5 (CPE CV-ICP OES)

[4]	Honey	0.5 g + 3 mL HNO_3 and 4 mL H_2O_2 were digested in the microwave oven (1400 W, 30 min) → volume completed to 50 mL high-purity water	30 min	CV-AFS	0.15 µg kg^{-1}	-	99%	1%	-
[5]	Honey	0.5 g sample + 4 mL HNO_3 and 2 mL H_2O_2 were digested in the microwave oven (1400 W, 30 min) → volume completed to 50 mL double deionised water	30 min	AMA	0.1 µg kg^{-1}	-	99%	-	3.7
[6]	Honey	For CV-AAS analysis: dilution in an acidic solution without heating. In the case of DMA, sample preparation was not necessary.	-	DMA or CV-AAS	1 or 30 µg kg^{-1} using DMA or CV-AAS, respectively	2.5 or 60 µg kg^{-1} using DMA or CV-AAS, respectively	CV-AAS: 98.7–102.4%; DMA: 95.6–99.1%	CV-AAS: 3.5–5.7%; DMA: 2.7–3.4%	1.3 or 1.5 for DMA or CV-AAS, respectively
[7]	Honey	1 g sample + 12 mL HNO_3 and 3 mL H_2O_2 were digested in the microwave oven	-	GF-AAS with a flow injection analysis system	-	-	91–113%	<10%	-
[8]	Bees, honey, and pollen	No sample pretreatment	-	AMA	0.5 µg kg^{-1}	-	-	-	-
[9]	Bees	bee were dried in a moisture analyzer to constant weight at 105 °C and ground → were digested in the microwave oven (45 min) → volume completed to 50 mL	45 min	ICP-OES	1 mg kg^{-1}	-	-	-	-
[10]	Honey, and pollen	0.5 g honey, and 0.3 g pollen + 7 mL HNO_3 and 2 mL H_2O_2 were digested in the microwave oven (from 180 to 240 °C, 40 min) → volume completed to 50 mL with deionized water.	40 min	ICP-MS	-	-	-	-	-
[11]	Beeswax, and honey	0.25 g beeswax + 5 mL HNO_3 and 1 mL H_2O_2 or 2 g honey + 5 mL HNO_3 were pre-digested at room temperature for 30 min, then digested using microwave oven (20 min)	50 min	High-Resolution ICP-OES	0.1 or 0.3 µg kg^{-1} for honey or beeswax, respectively	0.4 or 9 µg kg^{-1} for honey or beeswax, respectively	-	-	-

[12]	Pollen	0.65-0.70 g + 10 mL HNO_3 and 3 mL H_2O_2 were digested in the microwave oven →volume completed to 25 mL with a 5% HCl (v/v).	120 min	Hydride Generator coupled with ICP-OES	0.4 µg L^{-1}	2 µg L^{-1}	79–123%	6.4%	-
[13]	Propolis	~0.1 g; no sample pre-treatment	-	AMA-254	-	2 µg kg^{-1}	98%	<5.1%	2
[14]	Honey, and propolis	2 g of lyophilized samples + 5 mL HNO3 were heated at 50 °C for 2 h and then at 110 °C for 18 h; in the next step 5 mL H_2O_2 was added to the digested sample and was heated for 6 h → volume completed to 25 mL.	26 h	GF-AAS	-	-	-	-	-
[15]	Bees	0.7 g of lyophilized sample + 7 ml HNO_3 and 1.5 mL H_2O_2 were digested	-	DMA	-	10 µg kg^{-1}	-	-	-
[16]	Bees	~0.5 g of drieed sample + 7 ml HNO_3 and 2 mL H_2O_2 were digested in the microwave oven → volume completed to 25 mL with deionized water.	30 min	ICP-MS	10 µg kg^{-1}	-	94–108%	-	-

Table S2. Recovery and precision data for Hg in bees and beehive products (n = 3) by water bath digestion (95 °C, 30 min or 60 min).

		**Intermediate level spike (0.2 μg L^{-1})**				**High level spike (1 μg L^{-1})**			
		30 min		60 min		30 min		60 min	
**Matrix**	**mass (g)**	**R%**	**CV%**	**R%**	**CV%**	**R%**	**CV%**	**R%**	**CV%**
Honey	0.05	116	9.3	119	8.1	96	7.8	91	0.6
	0.1	104	14	115	4.8	96	3.0	92	4.0
	0.2	79	9.4	116	19	82	3.7	54	28
	1	48	52	71	9.7	82	8.9	18	2.8
Honeydew	0.05	121	6.5	113	4.1	101	3.5	94	4.9
	0.1	113	10	113	2.5	91	8.4	90	9.0
	0.2	107	21	114	2.8	82	1.5	92	2.6
	1	134	9.2	91	11	81	2.6	16	1.6
Pollen	0.02	90	3.7	110	5.9	95	3.6	84	1.4
	0.05	99	6.2	110	6.4	93	5.5	92	3.9
	0.1	116	5.7	113	4.6	91	5.7	91	5.5
	0.2	92	2.3	108	8.0	91	0.9	100	7.3
Propolis	0.02	98	8.6	109	9.3	91	2.5	80	3.9
	0.05	131	4.0	153	21	80	4.3	80	1.0
	0.1	161	15	145	19	55	26	74	37
	0.2	228	9.2	144	56	131	45	99	41
Beeswax	0.02	97	11	116	1.1	99	5.7	95	3.6
	0.05	111	8.5	112	0.7	99	2.0	93	6.1
	0.1	101	0.8	117	1.3	102	9.2	82	4.2
	0.2	110	13	108	0.5	76	39	89	5.0
Royal Jelly	0.02	108	4.4	111	5.8	110	0.9	95	2.0
	0.05	110	2.4	96	2.7	110	0.8	86	3.0
	0.1	100	2.8	87	6.0	101	3.2	89	7.7
	0.2	102	6.7	88	0.8	105	2.2	88	4.4
Bees	0.02	97	4.5	92	9.9	91	10	86	1.1
	0.05	99	4.1	105	8.9	97	9.8	80	0.1
	0.1	82	8.7	100	9.0	93	5.1	85	3.5
	0.2	108	7.8	99	0.7	98	7.0	89	3.6

References

1. Grainger, M.N.C.; Hewitt, N.; French, A.D. Optimised approach for small mass sample preparation and elemental analysis of bees and bee products by inductively coupled plasma mass spectrometry. *Talanta* **2020**, 214, 120858. doi: 10.1016/j.talanta.2020.120858.
2. Maragou, N.C.; Pavlidis, G.; Karasali, H.; Hatjina, F. Cold vapor atomic absorption and microwave digestion for the determination of mercury in honey, pollen, propolis and bees of greek origin. Global NEST J. 2016, 18(4), 690–696.
3. Dos Santos Depoi, F.; Bentlin, F.R.S.; Pozebon, D. Methodology for Hg determination in honey using cloud point extraction and cold vapour-inductively coupled plasma optical emission spectrometry. Anal. Methods 2010, 2, 180–185. doi: 10.1039/b9ay00189a.
4. Ru, Q.-M.; Feng, Q.; He, J.-Z. Risk assessment of heavy metals in honey consumed in Zhejiang province, southeastern China. Food Chem. Toxicol. 2013, 53, 256–262. doi: 10.1016/j.fct.2012.12.015.
5. Bilandžić, N.; Gačić, M.; Đokić, M.; Sedak, M.; Šipušić, Đ. I.; Končurat, A.; Gajger, I.T. (2014). Major and trace elements levels in multifloral and unifloral honeys in Croatia. J. Food Compos. Anal. 2014, 33, 132–138. doi: 10.1016/j.jfca.2013.12.002.
6. Vieira, H.P.; Nascentes, C.C.; Windmöller, C.C. Development and comparison of two analytical methods to quantify the mercury content in honey. J. Food Compos. Anal. 2014, 34, 1–6. doi: 10.1016/j.jfca.2014.02.001.
7. Meli, M.A.; Desideri, D.; Roselli, C.; Benedetti, C.; Feduzi, L. Essential and toxic elements in honeys from a region of central Italy. J. Toxicol. Env. Heal. 2015, A 78(10), 617–627. doi: 10.1080/15287394.2014.1004006.
8. Toth, T.; Kopernicka, M.; Sabo, R.; Kopernicka, T. The evaluation of mercury in honey bees and their products from eastern Slovakia. Scientific Papers: Animal Science and Biotechnologies 2016, 49, 257–260.
9. Dżugan, M.; Wesołowska, M.; Zaguła, G.; Kaczmarski, M.; Czernicka, M.; Puchalski, C. Honeybees (Apis mellifera) as a bio-logical barrier for contamination of honey by environmental toxic metals. Environ. Monit. Assess. 2018, 190, 101. doi: 10.1007/s10661-018-6474-0.
10. Jovetić, M.S.; Redžepović, A.S.; Nedić, N.M.; Vojt, D.; Đurđić, S.Z.; Brčeski, I.D.; Milojković-Opsenica, D.M. Urban honey - the aspects of its safety. Arh. Hig. Rada Toksikol. 2018, 69(3), 264–274. doi: 10.2478/aiht-2018-69-3126.
11. Bommuraj, V.; Chen, Y.; Klein, H.; Sperling, R.; Barel, S.; Shimshoni J.A. Pesticide and trace element residues in honey and beeswax combs from Israel in association with human risk assessment and honey adulteration. Food Chem. 2019, 299, 125123. doi: 10.1016/j.foodchem.2019.125123.
12. Morgano, M.A.; Martins, M.C.T.; Rabonato, L.C.; Milani, R.F.; Yotsuyanagi, K.; Rodriguez-Amaya, D.B. Inorganic contaminants in bee pollen from southeastern Brazil. J. Agr. Food Chem. 2010, 58, 6876–6883. doi: 10.1021/jf100433p.
13. Bonvehí, J.S.; Bermejo, F.J.O. Element content of propolis collected from different areas of South Spain. Environ. Monit. Assess. 2013, 185, 6035–6047. doi: 10.1016/j.foodchem.2019.125123.
14. Matin, G.; Kargar, N.; Buyukisik, H.B. Bio-monitoring of cadmium, lead, arsenic and mercury in industrial districts of Izmir, Turkey by using honey bees, propolis and pine tree leaves. Ecol. Eng. 2016, 90, 331–335. doi: 10.1016/j.ecoleng.2016.01.035.
15. Perugini, M.; Manera, M.; Grotta, L.; Abete, M.C.; Tarasco, R.; Amorena, M. Heavy metal (Hg, Cr, Cd, and Pb) contamination in urban areas and wildlife reserves: honeybees as bioindicators. *Biol. Trace Elem. Res.* **2011**, 140(2), 170–176. doi: 10.1007/s12011-010-8688-z.
16. Zaric, N.M.; Deljanin, I.; Ilijević, K.; Stanisavljević, L.; Ristić, M.; Gržetić, I. Assessment of spatial and temporal variations in trace element concentrations using honeybees (Apis mellifera) as bioindicators. *PeerJ* **2018**, 6:e5197. doi: 10.7717/peerj.5197.
